# Preparedness of Chinese nurses for emerging infectious diseases: evidence from a nationwide online cross-sectional survey on monkeypox knowledge and attitudes

**DOI:** 10.3389/fpubh.2026.1769762

**Published:** 2026-05-25

**Authors:** Yuan Gao, Fang-Yu Li, Yan-Fang Wang, Ling-Zhi Xu

**Affiliations:** 1 Department of Neurosurgery, Xuanwu Hospital, Capital Medical University, Beijing, China; 2 Innovation Center for Neurological Disorders and Department of Neurology, Xuanwu Hospital, Capital Medical University, Beijing, China; 3 Department of Gynecology, Affiliated Hospital of Shandong University of Traditional Chinese Medicine, Shandong, China

**Keywords:** China, cross-sectional survey, emerging infectious diseases, healthcare workforce, infection prevention and control, Mpox, nurses, preparedness

## Abstract

**Background:**

Emerging infectious diseases (EIDs) continue to pose recurrent challenges to global health systems. Nurses, as frontline healthcare professionals, play a pivotal role in early detection, infection control, and patient management. Mpox (monkeypox) outbreaks provide a real-world stress test for evaluating nurses’ preparedness for EIDs. This study assessed Chinese nurses’ knowledge and attitudes toward Mpox and examined training- and professional-related determinants of preparedness.

**Methods:**

A national online cross-sectional survey was conducted among nurses in China between January and March 2024 using convenience sampling. The questionnaire covered demographic characteristics, Mpox-related knowledge (27 items), and attitudes (7 items). Descriptive statistics, correlation analyses, and multivariable linear regression were performed.

**Results:**

A total of 387 valid responses were analyzed. Respondents were recruited from 24 provincial-level administrative regions, although the sample was concentrated in Eastern China. Overall, 81.9% of nurses demonstrated good Mpox-related knowledge, and 97.4% showed positive attitudes. Knowledge scores were positively correlated with attitudes (*p* < 0.001). Multivariable analysis revealed that professional title and receipt of Mpox-related workplace training were independently associated with higher knowledge scores (adjusted *R*^2^ = 9.0%). Professional title was also a significant determinant of attitudes (adjusted *R*^2^ = 3.9%).

**Conclusion:**

Chinese nurses exhibited generally high levels of knowledge and positive attitudes toward Mpox in the post-outbreak period, suggesting a high baseline awareness of EIDs. Workplace training and professional role differentiation appear central to preparedness. These findings highlight the importance of sustained, structured infectious disease education and leadership-oriented training to strengthen nursing preparedness for future EID threats.

## Introduction

Emerging infectious diseases (EIDs) remain a persistent and evolving threat to global public health, as demonstrated by outbreaks of COVID-19, avian influenza, and monkeypox (Mpox) (1–3). Such events repeatedly challenge healthcare systems, requiring rapid adaptation, effective infection prevention, and a well-prepared workforce. Among healthcare professionals, nurses constitute the largest group and are often the first point of contact with patients, positioning them at the forefront of disease recognition, infection control, and health education (4–6).

Mpox is a zoonotic viral disease characterized by fever, lymphadenopathy, and vesiculopustular rash, transmitted primarily through close contact (7, 8). Although historically confined to endemic regions in Africa, Mpox has caused multi-country outbreaks since 2022, prompting the World Health Organization to declare it a public health emergency of international concern (9, 10). China reported its first imported Mpox case in 2022 (11), followed by intensified surveillance, training, and response measures. In this context, Mpox represents not only a specific infectious threat but also a practical case through which healthcare system preparedness for EIDs can be evaluated.

Previous studies worldwide have assessed healthcare workers’ knowledge and attitudes toward Mpox, often reporting variable awareness and training gaps (12–14). In China, existing research has largely focused on mixed groups of healthcare workers, with limited attention to nurses as a distinct professional population (15, 16). Given nurses’ central role in infection prevention and patient care, understanding their preparedness—including knowledge, attitudes, and associated determinants—is critical for informing public health education strategies (5, 14, 17, 18).

Therefore, this study aimed to assess Chinese nurses’ knowledge and attitudes toward Mpox and to identify demographic and professional factors associated with preparedness. By framing Mpox as a representative EID scenario, the findings are intended to inform broader strategies for strengthening nursing education, leadership, and preparedness for future infectious disease threats.

## Methods

### Study design

A cross-sectional study was conducted from January to March 2024 to evaluate nurses’ knowledge and attitudes toward Mpox. The survey instrument was adapted and designed after reviewing relevant literature on Mpox (7) and consulting experts in the fields of public health and biology to align with the objectives of the study. To minimize potential bias, the survey was conducted anonymously, no personally identifiable information was collected, and the questionnaire was administered using a standardized format. This study is reported in accordance with the STROBE guidelines for cross-sectional studies (19).

### Sample, participants, and measures

Convenience sampling technique was selected for clinical nurses in China as the subjects for the survey. Several WeChat groups for nurses were sent a link to the questionnaire via Questionnaire Start and solicited the nurses who had already completed the questionnaire to refer their colleagues to participate in the survey. The online questionnaire was set to require completion of all items before submission.

#### Inclusion criteria

Possession of a valid Chinese nursing license; Age 18 or more; Informed consent and voluntary participation in this study; Be able to use a smartphone and participate in an online questionnaire survey.

#### Exclusion criteria

Lack of a valid nursing license or not registered in China; Not currently working in a healthcare institution, such as in academic or research institutions, administrative departments, or other areas not involving direct patient care; Inability to provide informed consent or refusal to participate in the study; Cognitive impairment or other conditions that affect the understanding of the study content and the provision of accurate feedback.

#### Sample size

The sample size was calculated using the formula $N=Z^{2}\times P\times (1-P)/d^{2}$ . Because the expected proportion of adequate Mpox-related knowledge among Chinese nurses was unknown, p was set at 0.50 to obtain the most conservative estimate. With a 95% confidence level, *Z* = 1.96, and a margin of error of 0.05 (20), the minimum analyzable sample size was 384. After allowing for a 10% non-response or invalid-response rate, the target sample size was 424.

### The instrument

The questionnaire consisted of three parts:

(1) Demographic variables with 11 items, including gender, age, marital status, region of work, education level, professional title, years of experience, hospital environment, department, experience working in isolation wards, and experience working in fever clinics (infectious disease departments). Region information was collected to describe the geographic coverage of the sample, but was not used for inferential subgroup analysis because the distribution across provinces was highly uneven.

(2) The nurses’ knowledge of Mpox, comprising 27 items assessing the level of understanding of Mpox among clinical nurses. All items were multiple-choice questions with “Yes,” “No,” and “Do not know” as options. Correct answers were awarded one point, while incorrect or “do not know” responses received no points. The total score which is defined as the sum of all 27 items, ranged from 0 to 27 points; 0–13 points were categorized as poor and 14–27 as good.

(3) The nurses’ attitudes toward Mpox, featuring 7 items to investigate the attitudes of clinical nurses toward Mpox. A 5-point Likert scale was used, with 1 point indicating strong disagreement, 2 points disagreement, 3 points neutral, 4 points agreement, and 5 points strong agreement. Scores ranged from 7 to 35 points, with less than 21 points indicating a negative attitude, and 21 points or more indicating a positive attitude.

Internal consistency was assessed in the present sample using Cronbach’s alpha for the knowledge section and Cronbach’s alpha for the attitude section. The internal consistency was acceptable, with a Cronbach’s alpha of 0.90 for the knowledge section and a Cronbach’s alpha of 0.76 for the attitude section.

### Ethical considerations

In accordance with local regulations, this anonymous questionnaire survey involving healthcare professionals did not collect any identifiable personal data and posed no more than minimal risk. Therefore, formal ethics committee approval was not required. The regulatory basis for this exemption is Article 32 of the Measures for Ethical Review of Life Science and Medical Research Involving Human Subjects (21), which stipulates that research using anonymized information data, without causing harm to subjects, and not involving sensitive personal information or commercial interests, may be exempted from ethical review. Before accessing the questionnaire, all participants provided electronic informed consent after being informed of the study’s purpose, voluntary nature, confidentiality, and their right to withdraw at any time before submission. The study was conducted in full compliance with the Declaration of Helsinki.

### Statistical analyses

The questionnaire was distributed via the Questionnaire Star platform. Characteristics data are expressed as means ± standard deviations (SD) for continuous variables and percentages for categorical variables. Correlations between scale scores were determined using Pearson’s correlation, and Mann–Whitney U tests, or one-way analysis of variance (ANOVA) were used to compare continuous variables between two or three and above groups. Univariate and multivariate linear regression were used to analyze the factors that influence Chinese Nurses’ Knowledge and Attitudes. The dependent variable in this study was the score of nurses’ knowledge or attitude toward Mpox. Variables associated with the outcome in univariate analyses at *p* < 0.05 were entered into the multivariable linear regression models. Regression coefficients, 95% confidence intervals, and *p* values were reported. IBM SPSS Statistics for Windows version 29 (IBM Corp., Armonk, NY, United States) was used for all statistical analyses. A two-tailed *p* < 0.05 was used as the threshold of statistical significance.

## Results

A total of 465 questionnaires were collected. After excluding those with arbitrary or patterned responses, 387 valid questionnaires remained, yielding an effective response rate of 83.2%, considered good (22).

### The general demographic characteristics

In the survey, 89.15% were females and males accounted for a smaller proportion of 10.85%. With respect to the healthcare facility levels, personnel from secondary or lower-tier hospitals constituted 10.85% of the respondents, whereas those from tertiary hospitals comprised a larger share of 89.15%. The majority of clinical nurses involved in the study were under 40, and most held a bachelor’s degree as their level of education. Additionally, 70.80% of the nurses reported having participated in training related to Mpox knowledge during their professional duties, and 58.91% of participants had isolation ward work experience (Table 1).

**Table 1:** Participant characteristics and knowledge and attitude scores toward the Mpox univariate analysis (*n* = 387).

Variables	*n*	%	Knowledge				Attitude			
			Mean	SD	Z/F	*P*	Mean	SD	Z/F	*P*
Gender					−0.48	0.63			−0.25	0.80
Male	42	10.85	19.38	5.76			30.40	4.31		
Female	345	89.15	19.07	5.38			30.79	3.57		
Age					9.16	<0.001			2.71	0.03
18–25	97	25.06	16.96	6.11			29.77	3.82		
26–30	186	48.06	19.52	5.24			31.02	3.39		
31–35	51	13.18	19.92	4.66			31.35	3.98		
36–40	32	8.27	21.31	4.48			31.41	3.44		
>40	21	5.43	20.00	3.58			30.33	4.09		
Marital status					−0.93	0.35			−0.77	0.44
Unmarried	217	56.07	18.87	5.54			30.64	3.64		
Married	170	43.93	19.40	5.25			30.88	3.68		
Education level					0.66	0.62			2.77	0.03
Secondary school	2	0.52	14.00	4.24			27.00	7.07		
Junior college	54	13.95	18.52	5.98			29.65	3.95		
Bachelor’s degree	304	78.55	19.24	5.38			30.99	3.59		
Master’s degree	24	6.20	19.04	4.79			30.13	3.23		
Doctorate	3	0.78	19.67	2.31			33.67	0.58		
Professional titles					8.27	<0.001			9.86	<0.001
Junior nurse	156	40.31	17.53	5.84			29.57	3.74		
Senior nurse	168	43.41	19.93	5.08			31.65	3.28		
Supervisor nurse	55	14.21	20.71	4.02			31.22	3.72		
Co-chief Superintendent Nurse or above	8	2.07	21.38	4.60			31.38	3.29		
Working experience					6.89	<0.001			2.30	0.06
<5	207	53.49	17.81	5.84			30.27	3.66		
5–10	102	26.36	20.41	4.72			31.20	3.60		
11–15	48	12.40	20.92	4.27			31.44	3.55		
16–20	16	4.13	21.19	3.76			32.13	3.40		
>20	14	3.62	20.07	3.87			30.57	3.92		
Working environment					−0.68	0.49			−2.44	0.02
Secondary hospitals and below	42	10.85	18.43	5.79			29.45	3.83		
Tertiary hospital	345	89.15	19.19	5.37			30.90	3.61		
Department					2.17	0.02			2.56	<0.007
Internal medicine	80	20.67	19.11	5.03			30.66	3.38		
Surgery	104	26.87	19.26	5.16			31.24	3.68		
Gynaecology and obstetrics	27	6.98	19.30	5.23			30.30	3.85		
Pediatrics	16	4.13	19.19	5.86			29.81	3.12		
Emergency	57	14.73	17.00	5.71			29.09	3.90		
Infectious disease	10	2.58	24.00	4.92			33.30	1.34		
Outpatient	10	2.58	20.40	5.90			31.20	4.39		
Operating	14	3.62	19.86	5.39			31.14	3.57		
Intensive care unit	37	9.56	18.86	5.75			31.41	3.32		
Others	32	8.27	20.13	5.24			31.28	3.75		
Isolation ward work experience					−1.31	0.19			−0.58	0.56
No	159	41.09	18.72	5.51			30.86	3.66		
Yes	228	58.91	19.37	5.34			30.67	3.66		
Fever clinic (infectious disease department) work experience					−1.41	0.16			−0.44	0.66
No	225	58.14	18.80	5.47			30.79	3.74		
Yes	162	41.86	19.52	5.32			30.69	3.55		
Mpox knowledge training during school					−0.16	0.87			−0.30	0.76
No	185	47.80	19.02	5.60			30.84	3.54		
Yes	202	52.20	19.18	5.25			30.66	3.77		
Mpox knowledge training during working					−3.17	0.002			−1.80	0.07
No	113	29.20	17.52	6.19			30.07	4.14		
Yes	274	70.80	19.76	4.92			31.03	3.41		

### Geographic distribution of participants

**Figure 1:**
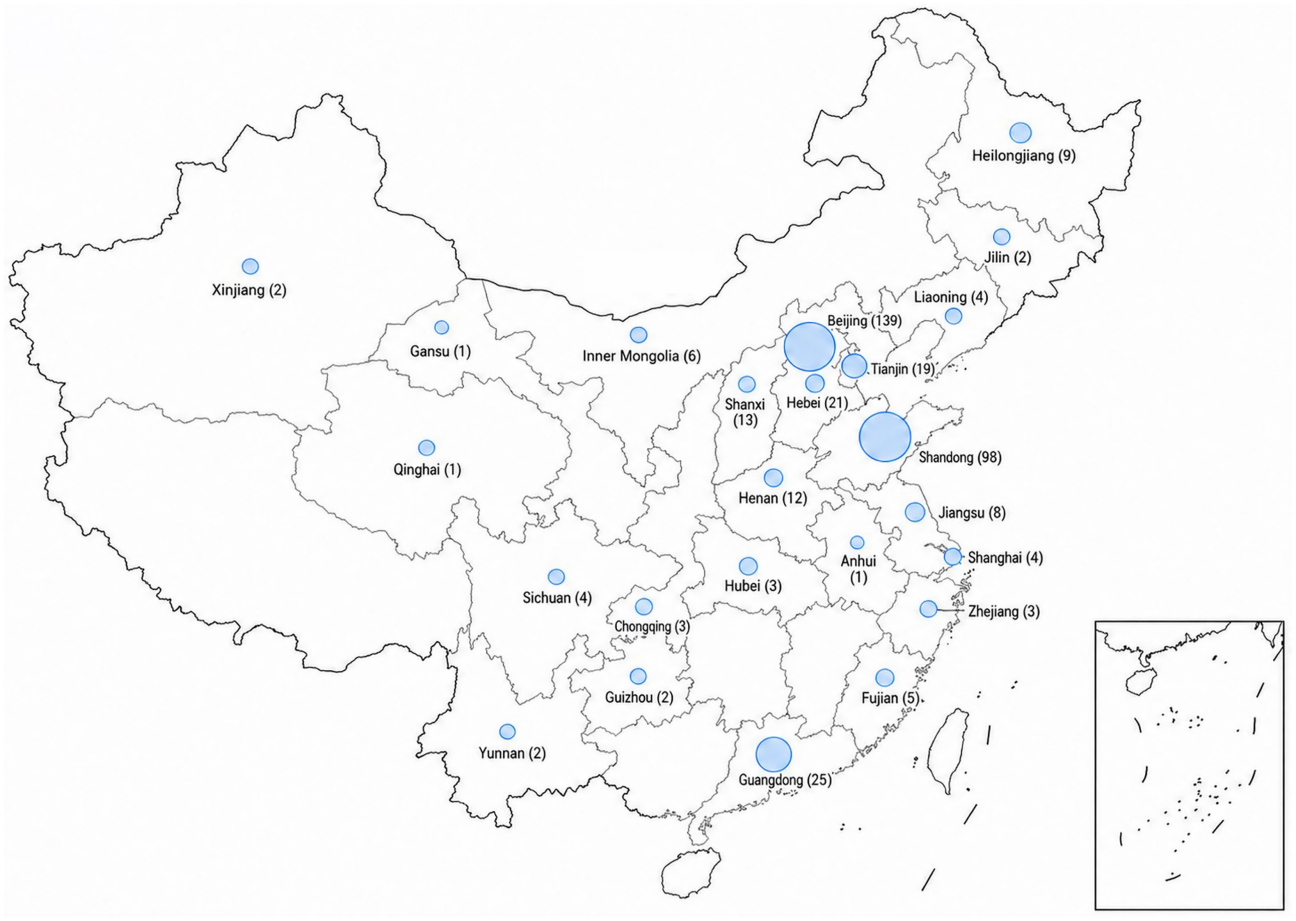
Geographic distribution of surveyed nurses in China. The map shows the distribution of the 387 surveyed nurses across provincial-level administrative regions in China. Circle size reflects the number of respondents in each province, municipality, or autonomous region. The figure is presented to describe the geographic coverage of the survey. Because the distribution of participants was uneven and the sample size was small in several provinces, no formal comparative analyses by region were performed. Created by the authors using original survey data. The China base map was adapted from the Standard Map Service System of the Ministry of Natural Resources of the People’s Republic of China (36).

Participants were recruited from multiple provincial-level administrative regions across China. The province-level distribution of respondents is shown in Figure 1 and Supplementary Table 2. Because the distribution was uneven and sample sizes were small in several provinces, no formal comparative analyses by region were performed.

### Knowledge and attitude

Overall, 81.91% of participants demonstrated good knowledge (Table 2). Most nurses exhibited a favorable attitude toward Mpox, constituting 97.42% of all participants (Table 3). Nurses’ knowledge was significantly positively correlated with attitudes (*r* = 0.05, *p* < 0.001). The participants’ responses to the knowledge and attitude questionnaire items are presented in Tables 4, 5.

**Table 2:** Distribution of nurses’ knowledge among Mpox.

Variables (*n* = 387)	*n*	%
Good knowledge	317	81.91
Poor knowledge	70	18.09

**Table 3:** Distribution of attitudes toward Mpox among nurses.

Variables (*n* = 387)	*n*	%
Positive attitude	377	97.42
Negative attitude	10	2.58

**Table 4:** Mpox knowledge scale (*n* = 387).

Questions	Answers					
	Correct		Wrong		Does not know	
	*n*	%	*n*	%	n	%
1. Are there any human monkeypox cases in China? (TRUE)	311	80.36	33	8.53	43	11.11
2. Monkeypox is prevalent in Africa. (TRUE)	302	78.04	33	8.53	52	13.44
3. Are there any human monkeypox cases in USA/Canada/UK/Europe? (TRUE)	305	78.81	22	5.68	60	15.50
4. Monkeypox is a viral disease infection? (TRUE)	326	84.24	33	8.53	28	7.24
5. Monkeypox is a bacterial disease infection? (FALSE)	251	64.86	101	26.10	35	9.04
6. Monkeypox is easily transmitted from human to human? (TRUE)	289	74.68	71	18.35	27	6.98
7. Monkeypox can be transmitted through the bite of an infected monkey. (TRUE)	260	67.18	74	19.12	53	13.70
8. Monkeypox and smallpox have similar signs and symptoms. (TRUE)	305	78.81	40	10.34	42	10.85
9. Monkeypox and smallpox have the same signs and symptoms. (FALSE)	138	35.66	190	49.10	59	15.25
10. The incubation period (time from infection to symptoms) for monkeypox is usually, 7–14 days but can range from 5 to 21 days. (TRUE)	292	75.45	40	10.34	55	14.21
11. Monkeypox illness typically lasts for 2–4 weeks. (TRUE)	268	69.25	47	12.14	72	18.60
12. Monkeypox virus can spread when a person comes into contact with the virus from, an infected animal. (TRUE)	286	73.90	56	14.47	45	11.63
13. Monkeypox virus can be spread when a person comes into contact with the virus from an infected person. (TRUE)	332	85.79	37	9.56	18	4.65
14. Monkeypox virus can spread through materials contaminated with the virus. (TRUE)	303	78.29	43	11.11	41	10.59
15. Monkeypox virus can cross the placenta from the mother to her fetus. (TRUE)	226	58.40	87	22.48	74	19.12
16. Monkeypox virus may also be spread through direct contact with body fluids or sores on an infected person or with materials that have touched body fluids or sores, such as clothing or linens. (TRUE)	314	81.14	36	9.30	37	9.56
17. Monkeypox can spread during intimate contact between people, including during sex and activities like kissing, cuddling, or touching parts of the body with monkeypox sores. (TRUE)	318	82.17	41	10.59	28	7.24
18. A flu-like syndrome is one of the human monkeypox’s early signs or symptoms. (TRUE)	286	73.90	50	12.92	51	13.18
19. Rashes (an area of irritated or swollen skin) on the skin are one of the signs or symptoms of human monkeypox. (TRUE)	277	71.58	73	18.86	37	9.56
20. Papules (which look like tiny, raised bumps on the skin) on the skin are one of the signs or symptoms of human monkeypox. (TRUE)	278	71.83	65	16.80	44	11.37
21. Vesicles (a thin-walled sac filled with a fluid, usually clear and small) on the skin are one of the signs or symptoms of human monkeypox. (TRUE)	274	70.80	60	15.50	53	13.70
22. Pustules (a bulging patch of skin full of a yellowish fluid called pus) on the skin are one of the signs or symptoms of human monkeypox. (TRUE)	286	73.90	52	13.44	49	12.66
23. Lymphadenopathy (enlargement of one or more lymph nodes) is one clinical sign or symptom that could be used to differentiate between monkeypox and smallpox cases. (TRUE)	270	69.77	45	11.63	72	18.60
24. One management option for symptomatic monkeypox patients is to use paracetamol. (TRUE)	216	55.81	65	16.80	106	27.39
25. Antivirals are required in the management of human monkeypox patients. (TRUE)	308	79.59	37	9.56	42	10.85
26. Antibiotics are required in the management of human monkeypox patients. (FALSE)	115	29.72	214	55.30	58	14.99
27. Diarrhea is one of the signs or symptoms of human monkeypox. (TRUE)	257	66.41	63	16.28	67	17.31

**Table 5:** Nurse attitude toward Mpox scale (*n* = 387).

Attitudes	Answers									
	Strongly disagree		Disagree		Neutrality		Agree		Strongly agree	
	*n*	%	*n*	%	*n*	%	*n*	%	*n*	%
1. I will avoid contacting animals that could harbor the virus.	4	1.03	3	0.78	22	5.68	95	24.55	263	67.96
2. I am ready to deal with monkeypox infected patients as a frontline fighter.	22	5.68	41	10.59	106	27.39	100	25.84	118	30.49
3. I am confident in maintaining standard precautions to prevent monkeypox virus transmission.	4	1.03	14	3.62	70	18.09	122	31.52	177	45.74
4. Adequate information related to the monkeypox virus is essential for nurses.	2	0.52	5	1.29	28	7.24	111	28.68	241	62.27
5. If I get infected, I will adequately maintain medical advice/isolation.	1	0.26	4	1.03	16	4.13	77	19.90	289	74.68
6. Appropriate nursing care with proper patient handling is crucial to prevent monkeypox virus transmission from patient to patient or patient to employee/attendant.	1	0.26	7	1.81	29	7.49	94	24.29	256	66.15
7. Nurses’ proper counseling to patients and attendants can reduce the viral disease prevalence rate.	0	0.00	4	1.03	23	5.94	89	23.00	271	70.03

### Univariate analysis of factors influencing Chinese nurses’ knowledge and attitudes toward Mpox

The nurses’ knowledge and attitude toward the monkeypox virus were considered dependent variables, while their participant characteristics were regarded as independent variables.

#### Knowledge

The study results revealed statistically significant differences in Mpox knowledge mastery among nurses based on age, professional titles, working experience, department and Mpox knowledge training during working (*p* < 0.05) (Table 1). Pairwise comparisons indicated that nurses aged 36–40 exhibited higher Mpox knowledge scores than other age groups. Furthermore, nurses holding the title of Co-chief Superintendent Nurse or above demonstrated a superior understanding of Mpox compared to their counterparts. Nurses with 16–20 years of working experience achieved the highest scores in terms of their knowledge about Mpox. Notably, those working in infectious disease departments displayed the most comprehensive understanding of Mpox. Additionally, nurses who received specific training on Mpox at work outperformed those who did not receive training.

#### Attitude

The variables of age, education level, professional title, working environment, and department demonstrated a statistically significant association with nurses’ attitudes toward Mpox (Table 1). Nurses aged 36 to 40 years showed a more positive attitude toward Mpox compared to other age groups. Nurses with higher educational qualifications, such as doctorate degrees, exhibited more positive attitudes toward Mpox than those with master’s, bachelor’s or associate degrees. The professional title of “Senior Nurse” was associated with the most positive attitudes toward Mpox. Additionally, working in tertiary hospitals nurses displayed significantly more favorable attitudes toward Mpox than those employed in secondary hospitals or lower. Among various departments, nurses in the infection department demonstrated the highest levels of positive attitudes toward Mpox.

### Multivariate regression analysis of factors influencing Chinese nurses’ knowledge and attitudes toward Mpox

Multiple regression analysis for the score of nurses’ knowledge of Mpox revealed that both nurses’ job title and whether they had received Mpox training at work significantly contributed to explaining their knowledge of Mpox, accounting for 9.5% of the total variance (Table 6). While the nurses’ attitude scores toward Mpox were utilized as the dependent variable, the results indicated that nurses’ job titles had a strong explanatory power for their attitude toward Mpox, accounting for 4.2% of the total variability (Table 7).

**Table 6:** Nurses’ knowledge scores multiple regression model.

Model	*B*	Std.	Beta	*t*	*P*	95% CI	Collinearity statistics	
							Tolerance	VIF
Constant	14.31	0.802		17.842	<0.001	(12.773, 15.887)		
Professional titles	1.737	0.345	0.245	5.031	<0.001	(1.058, 2.415)	0.997	1.003
Mpox knowledge training	2.403	0.578	0.202	4.158	<0.001	(1.267,3.540)	0.997	1.003

*R*^2^ = 9.5%, Adjusted *R*^2^ = 9% *F* = 17.286 *p* < 0.001.

**Table 7:** Nurses’ attitude scores toward Mpox multiple regression model.

Model	*B*	Std.	Beta	*t*	Sig	95% CI	Collinearity statistics	
							Tolerance	VIF
Constant	29.005	0.463		62.605	<0.001	(28.094, 29.916)		
Professional titles	0.978	0.239	0.204	4.089	<0.001	(0.508, 1.449)	1	1

*R*^2^ = 4.2%, Adjusted *R*^2^ = 3.9% *F* = 16.720 *p* < 0.001.

## Discussion

This nationwide online survey suggests that Chinese nurses showed generally high levels of Mpox-related knowledge and overwhelmingly positive attitudes during the post-outbreak period. These findings are consistent with recent studies conducted among healthcare workers in China and other countries, which reported improved awareness following targeted training and public health communication (15, 16, 23). These findings may reflect that there has been a consistent level of awareness and understanding about Mpox among healthcare professionals in China, which is an important component of preparedness for disease prevention and control measures.

Notably, the high prevalence of positive attitudes suggests a potential ceiling effect, indicating that attitudinal measures alone may have limited discriminatory power in post-outbreak contexts. Similar ceiling effects have been reported in post-epidemic KAP studies, where heightened risk perception and policy attention temporarily elevate attitude scores (24–26).

The variables that significantly influenced the Mpox knowledge among nurses in the multiple regression model were the Mpox knowledge training provided at their workplace and their professional title. Previous studies have revealed an alarming lack of knowledge regarding Mpox among healthcare workers before the outbreak in China, emphasizing the urgency for comprehensive training on this disease within healthcare (27). Workplace training emerged as a key determinant of knowledge, underscoring the importance of structured and timely educational interventions. Converging studies have consistently demonstrated that targeted infectious disease training significantly improves healthcare workers’ knowledge, confidence, and compliance with infection prevention practices (13, 25, 28, 29).

The professional title was associated with both knowledge and attitudes, potentially reflecting differences in leadership roles, clinical responsibilities, and involvement in infection control decision-making. A recent study further highlights the importance of advanced professional title nurses by demonstrating that they possess better mental resilience and stress management skills compared to those with lower professional titles (30). Senior nurses often serve as supervisors and role models, shaping both formal protocols and informal norms within clinical teams (31–33).

By conceptualizing Mpox as a stress test for EID preparedness rather than an isolated disease, this study contributes to a broader understanding of how nursing education and professional structure influence readiness for future public health emergencies. Similar preparedness-oriented frameworks have been advocated in recent public health and nursing literature (34, 35).

## Strengths and limitations

This study focused specifically on nurses, addressing a gap in the existing literature on healthcare worker preparedness for emerging infectious diseases in China. However, several limitations should be acknowledged. First, the use of convenience and snowball sampling, together with the cross-sectional design, may limit the generalizability of the findings and preclude causal inference. Although province information was collected, the geographic distribution of participants was uneven, with small sample sizes in several provinces; therefore, region-based comparative analyses were not performed, and the findings should not be interpreted as nationally representative. Second, city-level information and urban–rural classification were not collected, which prevented further analysis of whether participants worked in large urban centers or non-urban settings. Third, self-reported measures may overestimate actual preparedness because of social desirability bias. Finally, this study assessed knowledge and attitudes but did not directly evaluate clinical competence, infection-control behavior, or patient outcomes. Future studies employing broader sampling strategies and more balanced geographic recruitment are warranted.

## Conclusion

Chinese nurses show high baseline awareness and positive attitudes toward Mpox, reflecting substantial preparedness for emerging infectious diseases. Workplace training and professional role differentiation play central roles in shaping this preparedness. Sustainable, leadership-oriented public health education strategies are essential to equip nurses for future infectious disease challenges.

## Data Availability

The original contributions presented in the study are included in the article/Supplementary material, further inquiries can be directed to the corresponding author.
