# Supplementary material for: Preparedness of Chinese nurses for emerging infectious diseases: evidence from a nationwide online cross-sectional survey on monkeypox knowledge and attitudes
Source: Front Public Health. 2026 May 25;14:1769762. doi: 10.3389/fpubh.2026.1769762 (PMC13243426; doi:10.3389/fpubh.2026.1769762)
Supplement: Supplementary file 1 [file Table_1.DOCX]

**Supplementary Martials**

**Supplementary Table 1. The Methods of Valuation for Independent Variable**

| The Methods of Valuation for Independent Variable | |
| --- | --- |
| Independent Variable | **Valuation** |
| 1. Gender | Male=1, Female=2 |
| 2. Age (yr) | “18-25”=1, “26-30”=2, “31-35”=3, “36-40”=4, “＞40”=5 |
| 3. Marital status | Unmarried=1, Married=2 |
| 4. Educational level | Secondary School=1, Junior College=2, Bachelor’s Degree=3, Master’s Degree=4, Doctorate=5 |
| 5. Professional titles | Junior Nurse=1, Senior Nurse=2, Supervisor Nurse=3, Co-chief Superintendent Nurse or above=4 |
| 6. Working experience (yr) | “＜5”=1, “5-10”=2, “11-15”=3, “16-20”=4, “＞20”=5 |
| 7. Working environment | Secondary hospitals and below=0,  Tertiary hospital=1 |
| 8. Department | \| Internal medicine=1, \| \| --- \| \| Surgery=2, \| \| Gynaecology and obstetrics=3, \| \| Paediatrics=4, \| \| Emergency=5, \| \| Infectious Disease=6, \| \| Outpatient=7, \| \| Operating=8, \| \| Intensive care unit=9, \| \| Others=10 \| |
| 9. Isolation ward work experience | No=0, Yes=1 |
| 10. Fever clinic (infectious disease department) work experience | No=0, Yes=1 |
| 11. MopX knowledge training during school | No=0, Yes=1 |
| 12. MopX knowledge training during work | No=0, Yes=1 |

**Supplementary Table 2. Participants and Proportion by Region of Work**

| Participants and Proportion by Region of Work | | |
| --- | --- | --- |
| Province of work | **n** | **%** |
| Anhui | 1 | 0.26% |
| Beijing | 139 | 35.92% |
| Fujian | 5 | 1.29% |
| Gansu | 1 | 0.26% |
| Guangdong | 25 | 6.46% |
| Guizhou | 2 | 0.52% |
| Hebei | 21 | 5.43% |
| Henan | 12 | 3.10% |
| Heilongjiang | 9 | 2.33% |
| Hubei | 3 | 0.78% |
| Jilin | 2 | 0.52% |
| Jiangsu | 8 | 2.07% |
| Liaoning | 4 | 1.03% |
| Inner Mongolia | 6 | 1.55% |
| Qinghai | 1 | 0.26% |
| Shandong | 98 | 25.32% |
| Shanxi | 13 | 3.36% |
| Shanghai | 4 | 1.03% |
| Sichuan | 4 | 1.03% |
| Tianjin | 19 | 4.91% |
| Xinjiang | 2 | 0.52% |
| Yunnan | 2 | 0.52% |
| Zhejiang | 3 | 0.78% |
| Chongqing | 3 | 0.78% |

**Supplementary Table 3. STROBE Checklist**

| **STROBE Statement—Checklist of items that should be included in reports of cross-sectional studies** | | | |
| --- | --- | --- | --- |
|  | Item No | Recommendation | Reported on section |
| Title and abstract | 1 | (*a*) Indicate the study’s design with a commonly used term in the title or the abstract | Title page; Abstract |
|  |  | (*b*) Provide in the abstract an informative and balanced summary of what was done and what was found | Abstract |
| Introduction | | |  |
| Background/rationale | 2 | Explain the scientific background and rationale for the investigation being reported | Introductiom |
| Objectives | 3 | State specific objectives, including any prespecified hypotheses | Introduction, final paragraph |
| Methods | | |  |
| Study design | 4 | Present key elements of study design early in the paper | Methods – Study design |
| Setting | 5 | Describe the setting, locations, and relevant dates, including periods of recruitment, exposure, follow-up, and data collection | Methods – Study design; Sample and participants |
| Participants | 6 | (*a*) Give the eligibility criteria, and the sources and methods of selection of participants | Methods – Sample, participants, and measures |
| Variables | 7 | Clearly define all outcomes, exposures, predictors, potential confounders, and effect modifiers. Give diagnostic criteria, if applicable | Methods – The instrument; Statistical analyses |
| Data sources/ measurement | 8* | For each variable of interest, give sources of data and details of methods of assessment (measurement). Describe comparability of assessment methods if there is more than one group | Methods – The instrument |
| Bias | 9 | Describe any efforts to address potential sources of bias | Methods – Study design; Discussion – Limitations |
| Study size | 10 | Explain how the study size was arrived at | Methods – Sample size |
| Quantitative variables | 11 | Explain how quantitative variables were handled in the analyses. If applicable, describe which groupings were chosen and why | Methods – The instrument; Statistical analyses |
| Statistical methods | 12 | (*a*) Describe all statistical methods, including those used to control for confounding | Methods – Statistical analyses |
|  |  | (*b*) Describe any methods used to examine subgroups and interactions | N/A |
|  |  | (*c*) Explain how missing data were addressed | Methods – mandatory-response questionnaire |
|  |  | (*d*) If applicable, describe analytical methods taking account of sampling strategy | N/A |
|  |  | (*e*) Describe any sensitivity analyses | N/A |
| Results | | |  |
| Participants | 13* | (a) Report numbers of individuals at each stage of study—eg numbers potentially eligible, examined for eligibility, confirmed eligible, included in the study, completing follow-up, and analysed | Results – Participants |
|  |  | (b) Give reasons for non-participation at each stage | N/A |
|  |  | (c) Consider use of a flow diagram | Recommended to add figure |
| Descriptive data | 14* | (a) Give characteristics of study participants (eg demographic, clinical, social) and information on exposures and potential confounders | Results – Table 1 |
|  |  | (b) Indicate number of participants with missing data for each variable of interest | N/A |
| Outcome data | 15* | Report numbers of outcome events or summary measures | Results – Tables 2–5 |
| Main results | 16 | (*a*) Give unadjusted estimates and, if applicable, confounder-adjusted estimates and their precision (eg, 95% confidence interval). Make clear which confounders were adjusted for and why they were included | Results – Tables 6–7 |
|  |  | (*b*) Report category boundaries when continuous variables were categorized | Methods – scoring description; Table 1 |
|  |  | (*c*) If relevant, consider translating estimates of relative risk into absolute risk for a meaningful time period | N/A |
| Other analyses | 17 | Report other analyses done—eg analyses of subgroups and interactions, and sensitivity analyses | N/A |
| Discussion | | |  |
| Key results | 18 | Summarise key results with reference to study objectives | Discussion |
| Limitations | 19 | Discuss limitations of the study, taking into account sources of potential bias or imprecision. Discuss both direction and magnitude of any potential bias | Discussion – Strengths and limitations |
| Interpretation | 20 | Give a cautious overall interpretation of results considering objectives, limitations, multiplicity of analyses, results from similar studies, and other relevant evidence | Discussion |
| Generalisability | 21 | Discuss the generalisability (external validity) of the study results | Discussion – Strengths and limitations |
| Other information | | |  |
| Funding | 22 | Give the source of funding and the role of the funders for the present study and, if applicable, for the original study on which the present article is based | Acknowledgments |

*Give information separately for exposed and unexposed groups.
